# LDH-assisted growth of FeCo bimetal-MOF nanorods for electrocatalytic oxygen evolution†

**DOI:** 10.1039/d2ra04871j

**Published:** 2022-09-05

**Authors:** Lin Tang, Minjuan Cai, Maosheng Zhang, Xi Chen, Zhixiong Cai

**Affiliations:** College of Chemistry, Chemical Engineering and Environment, Minnan Normal University Zhangzhou 363000 China czx1816@mnnu.edu.cn; College of Chemistry and Chemical Engineering, Xiamen University Xiamen 361005 China

## Abstract

Metal–organic frameworks (MOFs) have emerged as alternative OER catalysts due to their easy regulation, such as *in situ* self-reconstruction from MOFs to metal hydroxides through alkaline hydrolysis. Herein, we demonstrate a facile strategy for the *in situ* transformation of FeCo layered double hydroxide (FeCo-LDH) nanosheets into 1D spindle-shaped FeCo-MOFs for efficient OER. An optimized electrode of FeCo-MOF on a nickel foam (NF) was achieved by adjusting the addition of organic ligands and the reaction time in the hydrothermal reaction. Based on the unique 1D nanostructure and the cation regulation, the obtained FeCo-MOF exhibits a good catalytic performance toward the OER with a low overpotential of 475 mV at 100 mA cm^−2^, a small Tafel slope of 121.8 mV dec^−1^, and high long-term durability. This study provides a facile strategy for preparing bimetal-MOFs as catalysts for efficient OER.

## Abstract 2

We demonstrate a strategy for transforming FeCo-LDH nanosheets into 1D FeCo-MOF nanorods *in situ* for efficient OER. This strategy solves the problem that MOFs are difficult to grow on conductive substrates under traditional synthesis conditions.

## Graphical abstract

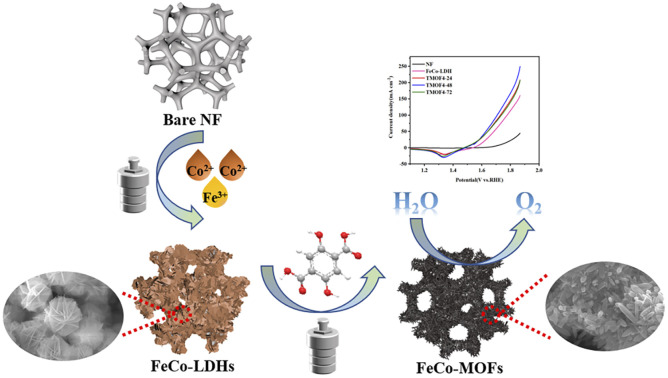

## Introduction

Owing to the rapid exhaustion of fossil energy and the concomitant environmental pollution, it is urgent to develop clean and renewable energy.^1,2^ Hydrogen has been widely regarded as the cleanest and ideal alternative to fossil fuels.^3,4^ One of the most appealing ways to produce hydrogen fuel is electrochemical water splitting, which comprises two half reactions, namely hydrogen evolution reaction (HER) and oxygen evolution reaction (OER).^5–8^ However, OER undergoes a four-electron transfer process and has a high reaction barrier, which is considered a rate-limiting step in thermodynamics and dynamics, limiting the efficiency of water electrolysis.^9–12^ To date, the most excellent catalysts for oxygen evolution are precious metal catalysts, such as ruthenium- and iridium-based oxides, but they cannot be used on a large scale owing to their low abundance and high price.^13–16^ Therefore, the development of efficient, stable, abundant and cheap non-noble metal catalysts for OER is of importance.

Among the developed non-noble metal catalysts, the Fe, Ni, Co based metal–organic frameworks (MOFs) have been widely used as catalysts in the OER process owing to their abundant catalytic active sites, large specific surface area, high porosity and adjustable nanostructure.^17,18^ Because of the synergistic effect between two different metal atoms in regulating the electronic performance of a catalyst, bimetallic MOF electrocatalysts have also attracted extensive attention. Huang *et al.* prepared a Fe/Ni bimetallic MOF using a one-step solvothermal method, and its excellent OER performance was mainly due to the unique structural characteristics of the MOF and the synergistic effect of metals. Xie *et al.* synthesized FeCo bimetal MOF nanosheets with excellent OER performance under alkaline conditions.^19^ However, the electrocatalytic properties of MOFs are limited by their poor electrical conductivity and low mass transfer efficiency.^20^ In addition, the powdered MOFs must be attached to electrodes with the assistance of binders before being applied to the electrochemical test, which would restack and reduce the original activity and mechanical stability of the catalysts.^21^ For direct utilization as OER electrocatalysts, growing MOFs directly on conductive substrates sites is a simple and effective strategy to address these issues, which possess closer contact and a more accessible active site for improving the electrical conductivity and mechanical stability of MOFs.^22,23^ However, the controllable growth of MOFs on conductive substrates remains a daunting challenge because MOFs tend to form bulk crystals under conventional synthetic conditions. Therefore, an effective synthesis strategy is required to directly grow MOFs on conductive substrates for the practical application of MOFs in electrocatalysis.

Moreover, 2D layered double hydroxides (LDHs) based on Fe, Ni, and Co metal ions have been studied extensively owing to their rich metal sites, multimetal tunability and good catalytic activity.^24^ In addition, 2D LDHs have reasonable and adjustable layer spacing, allowing the organic ligand to react with interlayer metal ions to generate MOFs.^25^ Thus, 2D LDHs are always chosen as precursors or sacrificial templates for the preparation of 2D MOF nanosheets or 1D MOF nanorods by regulating the synthetic conditions. The resultant 2D or 1D MOFs possess the expected highly exposed active sites, a large surface area, and enhanced conductivity in electrocatalysis. Unfortunately, the stability of these electrocatalysts during long-term OER was rarely mentioned despite progress in increasing their activity, as low-dimensional nanomaterials usually spontaneously aggregate to reduce their high specific surface energy.

Herein, robust spindle-like FeCo bimetal MOF nanorods (NDs) supported on a nickel foam substrate were fabricated *via* a sacrificial template-assisted growth strategy. Specifically, the pre-synthesized FeCo LDH serves as sacrificial precursors, which provide an appropriate dissolution rate of metal ions in the electrode vicinity, enabling the *in situ* transformation into FeCo-MOF upon adding the organic ligand. The obtained self-supported electrocatalyst exhibited good catalytic performance with a low overpotential of 475 mV at 100 mA cm^−2^ and a low Tafel slope of 121.8 mV dec^−1^. After 1000 CV cycles and a 12 h *i*–*t* test, the catalytic activity maintained high stability, with barely any change.

## Experiment

### Materials

2,5-Dihydroxyterephthalic acid (H_4_DOBDC, 98%), Co(NO_3_)_2_·6H_2_O (AR), FeCl_3_·6H_2_O (AR), NH_4_F and urea were obtained from Energy Chemical; *N*,*N*-dimethylformamide (DMF AR), hydrochloric acid (AR), ethanol (AR), and potassium hydroxide (AR) were purchased from Zhangzhou Cuilin Co., Ltd. NF was purchased from Kunshan Guangjiayuan New Materials Co., Ltd. All these chemicals were used without further purification.

### Synthesis of FeCo-LDH nanosheets

Typically, 0.0811 g FeCl_3_·6H_2_O (0.3 mmol), 0.4365 g Co(NO_3_)_2_·6H_2_O (1.5 mmol), 0.114 g NH_4_F (3 mmol) and 0.36 g urea (6 mmol) were dissolved into 25 mL deionized water and then transferred into a 50 mL Teflon-lined autoclave. Then, a piece of pretreated NF (1 cm × 3 cm) was added to the above solution. The autoclave was then heated at 120 °C for 8 hours. After cooling to room temperature, the collected product was washed several times with deionized water and then dried at 60 °C.

### Synthesis of FeCo-MOF nanorods

1 mL deionized water was added to 16 mL DMF containing 0.064 g 2,5-dihydroxyterephthalic acid (4 mg mL^−1^) and transferred to a 25 mL Teflon-lined autoclave. Then, a piece of FeCo-LDH/NF (1 × 3 cm) was immersed in the above solution and reacted at 120 °C for 48 hours. After cooling to room temperature, the collected product was washed several times with absolute ethanol and then dried at 60 °C. For comparison, samples with different coordination times (24 h and 72 h) and ligand concentrations (2 and 6 mg mL^−1^) were also prepared.

### Physical characterization

The morphologies and distribution of the elements in the as-prepared catalysts were characterized using a scanning electron microscope (SEM, ZEISS GeminiSEM 300). The SEM images were obtained at an acceleration voltage of 3 kV. The element mappings were obtained at an acceleration voltage of 20 kV. The fine microstructure of the as-prepared catalysts was characterized *via* transmission electron microscopy (TEM, FEI Talos F200x) at an acceleration voltage of 200 kV. Powder X-ray diffraction (XRD, Japan Rigaku Ultima IV) with Cu Kα radiation was used to characterize the crystalline phase of the as-prepared catalysts. X-Ray photoelectron spectroscopy (XPS) measurements were conducted using a Thermo Scientific K-Alpha spectrometer equipped with a monochromatic Al Kα radiation. All peaks were calibrated with the C 1s peak binding energy at 284.8 eV for adventitious carbon.

### Electrochemical measurements

All electrochemical experiments were carried out in a three-electrode system using a CHI 660E electrochemical workstation in 1 M KOH solution. The counter electrode is a platinum sheet, and the reference electrode is a calomel electrode with saturated KCl solution. The measured potentials were converted into reversible hydrogen electrode potentials using the following formula: *E*_RHE_ = *E*_SCE_ + 0.241 V + 0.059 pH. In order to avoid interference from the oxidation peak of nickel foam, linear sweep voltammetry tests were carried out from high potential to low potential at the scanning rate of 5 mV s^−1^. Electrochemical impedance spectroscopy (EIS) experiments were performed at 1.5 V, and the frequency range was 10^5^ to 0.1 Hz. In order to evaluate the electrochemical double-layer capacitances, cyclic voltammetry (CV) measurements were performed between 1.07 and 1.17 V (*vs.* RHE) at scan rates from 20 to 100 mV s^−1^. The stability tests were performed using amperometric *i*–*t* curves at the potential corresponding to the current density of approximately 10 mA cm^−2^ and cyclic voltammetry tests between 1.07 and 1.47 V (*vs.* RHE) for 1000 cycles at the scanning rate of 50 mV s^−1^.

## Results and discussion

### Preparation and characterization of 1D FeCo-MOFs

**Scheme 1:**
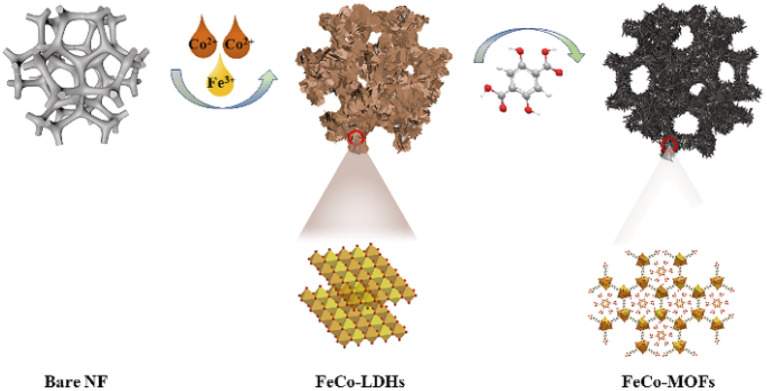
Schematic of the ligand-assisted transformation for the preparation of spindle-like FeCo-MOF NDs.

**Fig. 1:**
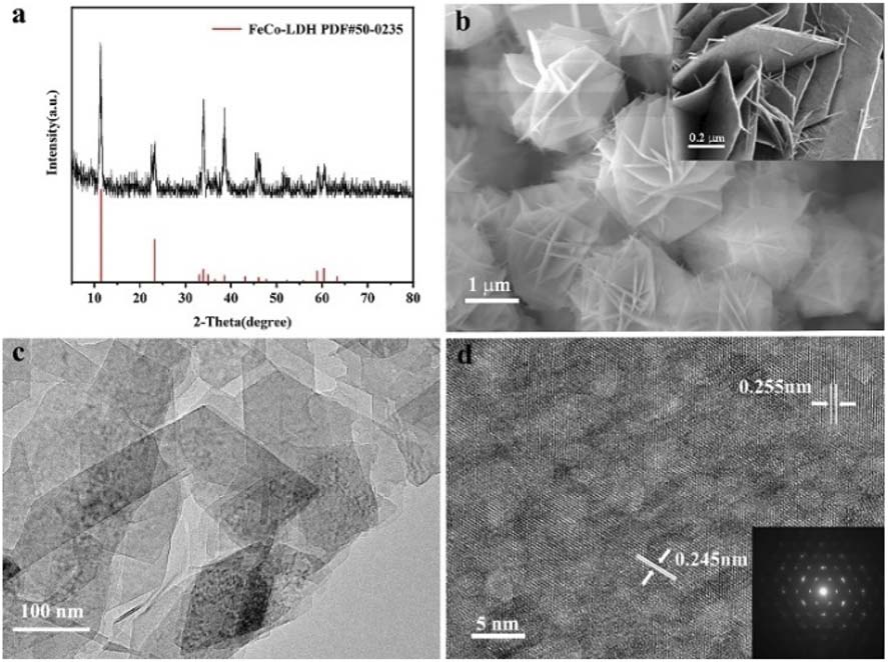
(a) XRD pattern, (b) SEM image, (c) TEM image and (d) HRTEM image of the FeCo-LDH.

**Fig. 2:**
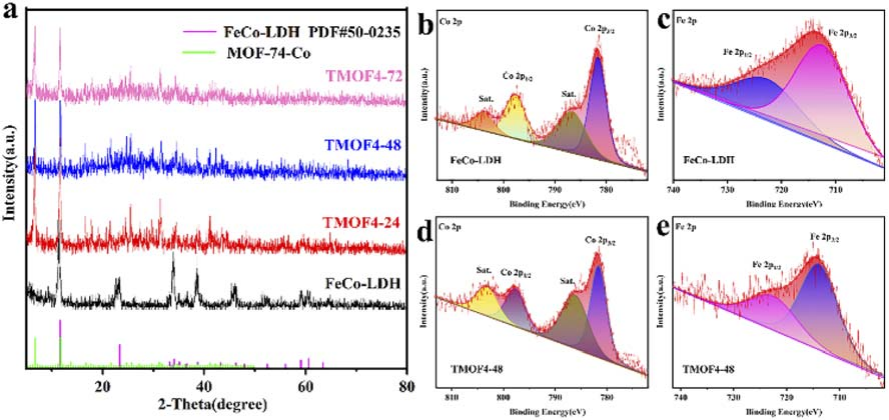
(a) XRD patterns of FeCo-LDH, TMOF4-24, TMOF4-48 and TMOF4-72. (b) XPS of Co 2p in FeCo-LDH. (c) XPS of Fe 2p for FeCo-LDH. (d) XPS of Co 2p of the TMOF4-48. (e) XPS of Fe 2p of the TMOF4-48.

The *in situ* transformation growth strategy is illustrated in Scheme 1, which involves the synthesis of the 2D FeCo-LDH and the subsequent ligand-assisted growth of FeCo MOFs. First, FeCl_3_·6H_2_O and Co(NO_3_)_2_·6H_2_O (the molar ratio of Fe : Co was 1 : 5) were used as metal sources to synthesize the 2D FeCo-LDH *via* a hydrothermal process. The X-ray powder diffraction (XRD) pattern of FeCo-LDH is shown in Fig. 1a. The peaks at 11.6°, 23.4°, 34.1°, 35.1°, 36.6°, 38.7°, 46.2°, 52.4°, 59.1° and 60.5°can be assigned to the (003), (006), (012), (009), (104), (015), (018), (1010), (110), and (113) facets of FeCo-LDH (PDF#50-0235), respectively, confirming the successful synthesis of FeCo-LDH. The SEM image (Fig. 1b) shows that the morphology of the synthesized FeCo-LDH is a petal-like structure composed of nanosheets, which is further confirmed by the TEM image (Fig. 1c). As shown in Fig. 1d, the lattice spacings are 0.245 nm and 0.255 nm, which correspond to the (104) and (009) crystal planes of FeCo-LDH, respectively. Moreover, the coexistence of Co^2+^ and Fe^3+^ ions in the FeCo-LDH nanosheets was also confirmed *via* high resolution X-ray photoelectron spectroscopy (XPS) (Fig. 2b and c). The spectra of Co 2p for FeCo-LDH show that the peaks at 781.6 eV and 797.7 eV can be assigned to Co 2p_3/2_ and Co 2p_1/2_, respectively, indicating the presence of Co^2+^. As shown in Fig. 2c, the binding energies of Fe 2p_3/2_ and Fe 2p_1/2_ at 712.5 eV and 722.6 eV, respectively, prove the existence of Fe^3+^. These observations prove that FeCo-LDH was successfully synthesized.

**Fig. 3:**
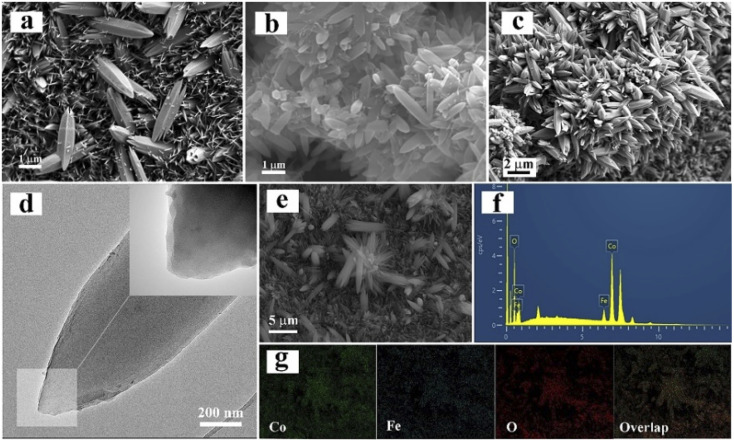
SEM images of (a) TMOF4-24, (b) TMOF4-48 and (c) TMOF4-72. (d) TEM images of TMOF4-48. (e–g) EDS and elemental mappings of TMOF4-48.

In the second step, 2,5-dihydroxyterephthalic acid was added and reacted with the previously prepared FeCo-LDH to generate FeCo-MOF *in situ*. In order to study the changes in the material morphology and composition during the reaction process, the materials prepared at different reaction times were tested using SEM and XRD. For convenience, the samples after the coordination reaction are denoted by TMOF_*X*–*Y*_, where *X* represents the concentration of the ligand and *Y* represents the coordination time. Therefore, the samples obtained after the reaction of 24, 48 and 72 h are denoted as TMOF4-24, TMOF4-48, and TMOF4-72, respectively. The SEM images of the samples based on the time-dependent reaction are displayed to examine the morphology evolution. As shown in Fig. 3a, a small number of one-dimensional spindle-shaped nanorods appeared and some small nanosheets remained, indicating that the morphology of the sample changed after reacting with the ligand for 24 hours. The XRD pattern of TMOF4-24 (Fig. 2a) shows that the diffraction peak intensity of FeCo-LDH is weakened and diffraction peaks belonging to Co-MOF appear, which confirms that the one-dimensional spindle-shaped nanorods in the SEM image of TMOF4-24 are FeCo-MOFs. As shown in Fig. 3b and c, with an increase in the reaction time, the number of spindle-shaped nanorods increases, while the nanosheets are almost invisible. As shown in Fig. 2a, only the Co-MOF but not the FeCo-LDH crystal phase can be observed in the XRD pattern of TMOF4-48. The same pattern can be observed in the XRD pattern of TMOF4-72. This shows that FeCo-LDH was converted to FeCo-MOF after 48 h of the reaction.

Based on the above-mentioned characterizations, we can infer that two main processes are involved in the transformation process: the dissolution of FeCo-LDH and the growth of FeCo-MOF. First, the metal ions in the FeCo-LDH dissolved gradually, making the nanosheets smaller. Simultaneously, the added organic ligand reacted with the released metal ions to form the FeCo-MOF until the FeCo-LDH nanosheets were completely exhausted.

In order to further study the transformation of the material morphology and composition during the reaction process, samples prepared with different ligand concentrations were characterized *via* SEM and XRD. As shown in Fig. S1b,† a small number of spindle-shaped nanorods that had not yet been fully grown appeared and some small nanosheets remained. The XRD pattern of TMOF2-48 (Fig. S2a†) shows diffraction peaks belonging to FeCo-LDH and Co-MOF, similar to TMOF4-24 (Fig. 2a). It can be observed from the SEM image of TMOF6-48 (Fig. S1d†) that there are many spindle-shaped nanorods, while the nanosheets are almost invisible. The Co-MOF but not the FeCo-LDH crystal phase can be observed in the XRD pattern of TMOF6-48 (Fig. S2a†), just like TMOF4-72 (Fig. 2a). These control experiments indicate that both reaction times and ligand concentrations affect the transformation of the crystalline phase.

In order to further reveal the microstructure, elemental composition and chemical states of TMOF4-48, TEM, SEM and XPS characterizations were further performed. Under the SEM results, the TEM image (Fig. 3d) shows the presence of 1D spindle-shaped nanorods. From the elemental mappings of TMOF4-48 (Fig. 3g), we can observe that Co, Fe and O are uniformly distributed throughout the sample. Moreover, the coexistence of Co^2+^ and Fe^3+^ ions in TMOF4-48 was also confirmed by the high resolution X-ray photoelectron spectroscopy (XPS) (Fig. 2d and e). The spectra of Co 2p for TMOF4-48 (Fig. 2d) show that the peaks at 781.6 eV and 797.7 eV can be assigned to Co 2p_3/2_ and Co 2p_1/2_, respectively, indicating the presence of Co^2+^. As shown in Fig. 2e, the binding energies of Fe 2p_3/2_ and Fe 2p_1/2_ at 712.5 eV and 722.6 eV, respectively, prove the existence of Fe^3+^.

### Electrocatalytic performance of the prepared 1D FeCo-MOFs toward the OER

**Fig. 4:**
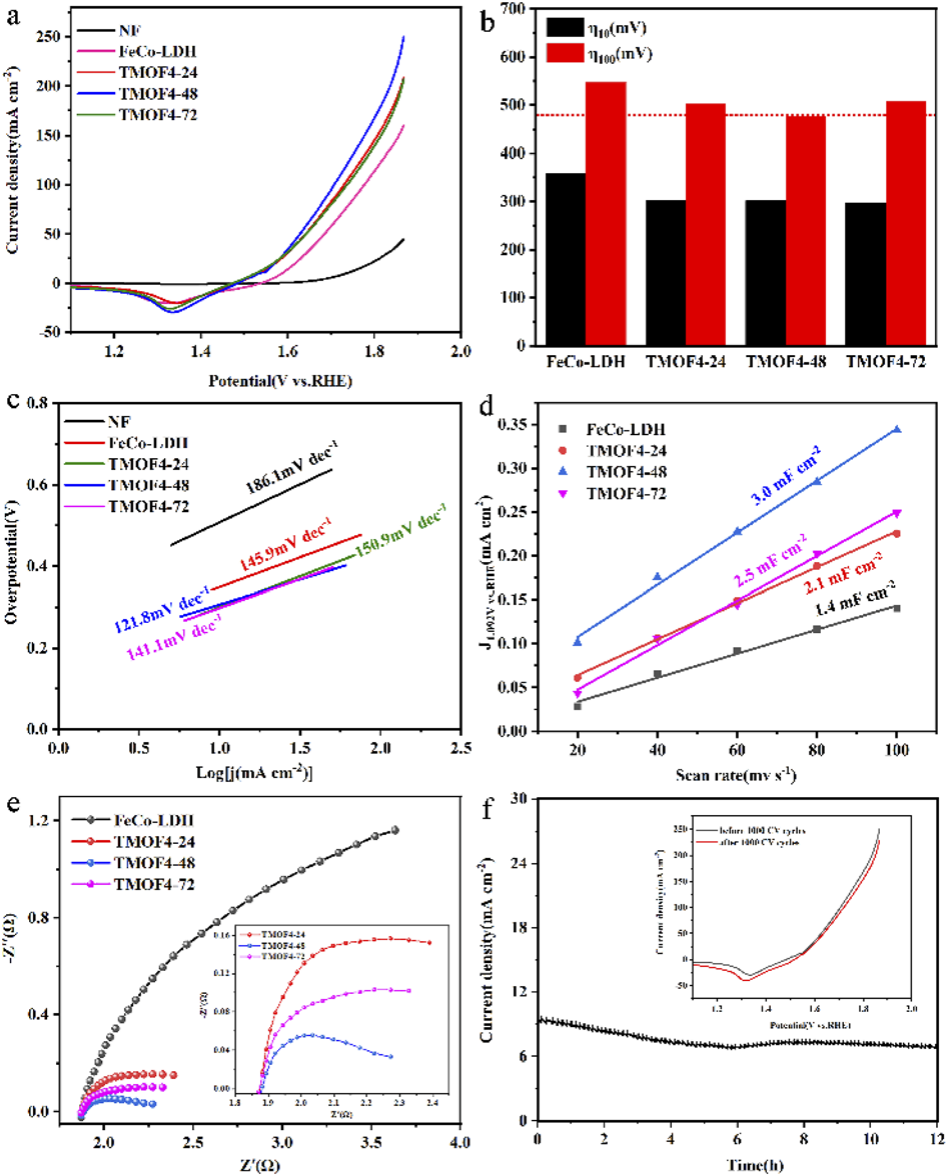
(a) Polarization curves of the OER on FeCo-LDH, TMOF4-24, TMOF4-48, TMOF4-72 and NF. (b) Comparison of the overpotentials at 100 mA cm^−2^ (*η*_100_) and 10 mA cm^−2^ (*η*_10_) obtained from the studied materials in 1.0 M KOH. (c) Tafel plots of OER for the studied materials. (d) *C*_dl_ values of the FeCo-LDH, TMOF4-24, TMOF4-48 and TMOF4-72. (e) EIS spectra of the prepared catalysts in 1.0 M KOH at 1.5 V. (f) Durability tests of TMOF4-48 in 1.0 M KOH; inset is the polarization curves before and after 1000 CV cycles.

The electrocatalytic OER performance of TMOF4-48/NF was evaluated in a three-electrode system using a CHI660E electrochemical workstation in 1 M KOH solution. For comparison, samples were prepared at different reaction times and ligand concentrations; FeCo-LDH and NF were tested under the same experimental conditions. As shown in Fig. 4a and b, NF exhibited the worst electrocatalytic performance.

When the current density reached 100 mA cm^−2^, the overpotentials required by the prepared MOFs were smaller than those of FeCo-LDH. The overpotential of TMOF4-48 is 475 mV, which is smaller than those of TMO4-24 (505 mV) and TMOF4-72 (507 mV), indicating that TMOF4-48 has the best electrocatalytic performance. Meanwhile, TMOF4-48 also has a low Tafel slope (121.8 mV dec^−1^), which is smaller than that of FeCo-LDH (145.9 mV dec^−1^), TMOF4-24 (150.9 mV dec^−1^) and TMOF4-72 (141.1 mV dec^−1^), indicating that it has the fastest oxygen evolution kinetics (Fig. 4c). Moreover, compared with TMOF2-48 (532 mV, 170.6 mV dec^−1^) and TMOF6-48 (512 mV, 161.9 mV dec^−1^), TMOF4-48 also has the smallest overpotential and Tafel slope, indicating that its electrocatalytic performance is the best (Fig. S3a and b†).

TMOF4-48 is superior to the electrochemically active surface area (ECSA). By conducting CV experiments at different sweep speeds (Fig. S4†), double-layer capacitance (*C*_dl_) was obtained, and *C*_dl_ was proportional to the electrochemically active surface area. Therefore, the ECSA of the samples can be evaluated by comparing the values of *C*_dl_.^26^ As shown in Fig. 4d and S3c,† the *C*_dl_ of TMOF4-48 (3.0 mF cm^−2^) is larger than those of the other samples.

This indicates that TMOF4-48 has the largest electrochemical active surface area and can provide more active sites in the electrocatalytic process, which is advantageous for improving electrocatalytic performance.

We also carried out EIS measurements on the samples to further reveal the reasons for the excellent electrocatalytic performance of TMOF4-48. As shown in Fig. 4e and S3d,† after adding the organic ligand, the diameter of the semicircle of the samples decreased, indicating that the charge transfer impedance of the samples decreased. Among them, the diameter of the semicircle of TMOF4-48 is the smallest, which means that it has the smallest charge transfer resistance, that is, the charge transfer rate is the fastest, which is conducive for improving the electrocatalytic performance.

**Fig. 5:**
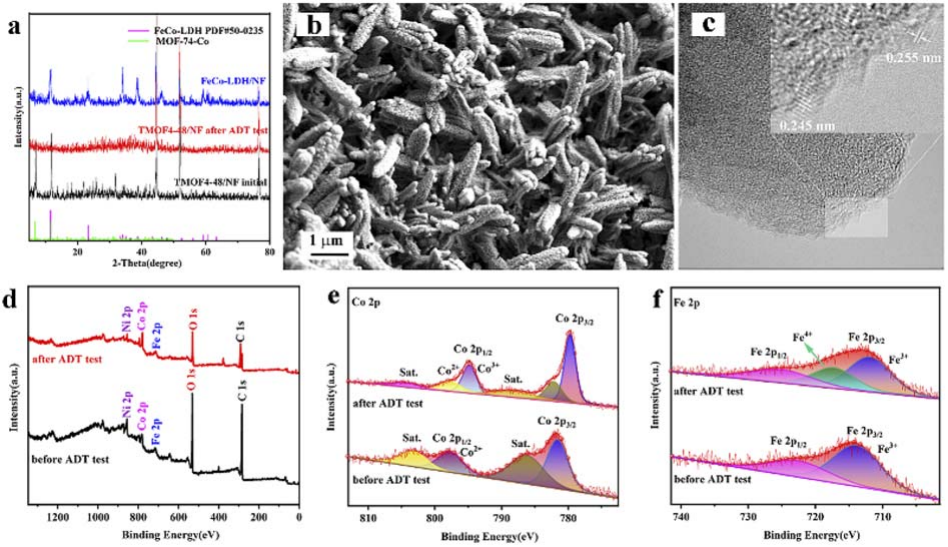
(a) XRD patterns of FeCo-LDH/NF, TMOF4-48/NF and TMOF4-48/NF after the long-term durability test. (b) SEM image of TMOF4-48 after the long-term durability test. (c) HRTEM image of TMOF4-48 after the long-term durability test. (d–f) XPS survey spectra and the corresponding Co 2p and Fe 2p spectra of TMOF4-48 before and after the long-term durability test.

Long-term durability is also an important parameter for evaluating the feasibility of catalysts in practical applications. The stability of the prepared TMOF4-48 was assessed using amperometric *i*–*t* curves and CV cycling tests. As shown in Fig. 4f, the current density of TMOF4-48 showed a small loss after 12 hours of the *i*–*t* test, demonstrating that TMOF4-48 has excellent stability under alkaline conditions. Moreover, as shown in Fig. 4f inset, after 1000 cycles of CV tests, the LSV curve slightly differs from that before, which further proves that it has excellent durability. In addition, SEM, XPS, TEM and XRD measurements were performed on the TMOF4-48 after the long-term durability test to observe the changes in its morphology, composition and chemical states. The SEM images (Fig. 5b) show that after the long-term durability test, the spindle-shaped nanorods were still maintained, but some agglomerated nanoparticles were present on the surface of the nanorods. The XPS results (Fig. 5d–f) show that the samples before and after the long-term durability test contained Co, Fe, O and C. Apart from Co^3+^ and Fe^4+^ generated by the partial oxidation of Fe and Co in the OER process, Co^2+^ and Fe^3+^ were mainly present in the samples after the test. Moreover, the HRTEM image (Fig. 5c) shows lattice spacings of 0.245 nm and 0.255 nm, which correspond to the (104) and (009) crystal planes of FeCo-LDH, respectively. These results indicate that part of the MOF phase may be reconverted to the LDH phase during the OER in alkaline electrolyte. However, after the long-term durability test, only the NF but not the MOF crystal phase can be observed in the XRD pattern (Fig. 5a). This may be because less active material was loaded onto the NF after a long period of testing. The above characterizations indicate that TMOF4-48 has good electrochemical stability and structural stability, but its mechanical stability is still not excellent.

## Conclusions

In summary, a facile strategy was developed for the *in situ* transformation of FeCo-layered double hydroxide nanosheets into 1D spindle-shaped FeCo-MOFs for efficient OER. As precursors, LDHs can provide metal ions and control the release rate of metal ions, which is conducive to the growth of MOFs. The morphology and catalytic properties of MOFs can be changed by adjusting their ligand concentration and the coordination reaction time. When the coordination time was 48 h and the ligand concentration was 4 mg mL^−1^, one-dimensional spindle-shaped MOF crystals were formed, which exhibited good catalytic performance. The optimal FeCo-MOF showed the lowest overpotential of 475 mV at 100 mA cm^−2^ and the smallest Tafel slope of 121.8 mV dec^−1^. The optimal FeCo-MOF had high electrocatalytic stability; after 1000 cycles of CV tests and 12 h of the *i*–*t* test, its catalytic activity changed insignificantly. The good electrocatalytic performance can be attributed to the unique structural characteristics of the prepared 1D FeCo-MOFs, providing abundant active sites. This is advantageous for improving electrocatalytic performance. This study provides a facile strategy for preparing non-noble metal catalysts for efficient OER. We believe that this strategy will be widely used in the preparation of MOF-based materials in the future.

## Conflicts of interest

There are no conflicts to declare.

## Supplementary Material

RA-012-D2RA04871J-s001
