# Supplementary material for: LDH-assisted growth of FeCo bimetal-MOF nanorods for electrocatalytic oxygen evolution
Source: RSC Adv. 2022 Sep 5;12(38):25112–7. doi: 10.1039/d2ra04871j (PMC9443477; doi:10.1039/d2ra04871j)
Supplement: RA-012-D2RA04871J-s001 [file RA-012-D2RA04871J-s001.pdf]

Electronic Supplementary Material (ESI) for RSC Advances.
This journal is © The Royal Society of Chemistry 2022

Supplementary Materials

LDH-assisted Growth of FeCo Bimetal-MOF Nanorods for Electrocatalytic Oxygen Evolution

Lin Tang[a], Minjuan Cai[a], Maosheng Zhang[a], Xi Chen[b], Zhixiong Cai[a*]

[a] *College of Chemistry, Chemical Engineering and Environment, Minnan Normal University, Zhangzhou 363000, China*

[b] *College of Chemistry and Chemical Engineering, Xiamen University, Xiamen 361005, China*

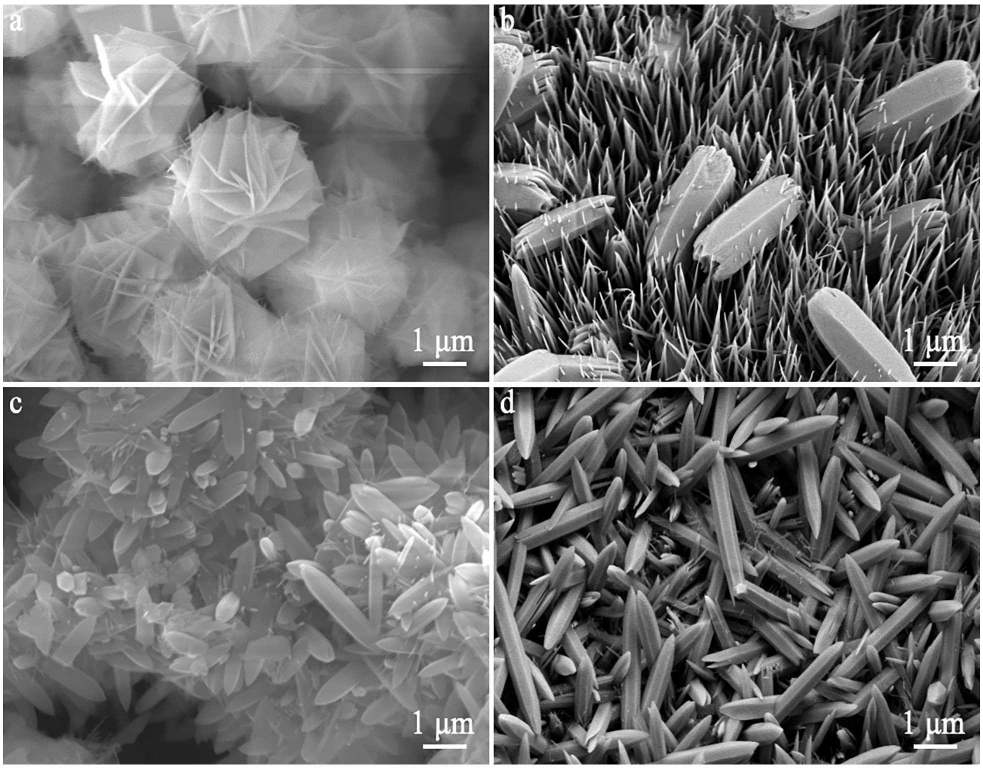

Fig. S1 SEM images of (a) FeCo-LDH, (b) TMOF2-48, (c) TMOF4-48 and (d) TMOF6-48.

* Corresponding author.
E-mail addresses: czx1816@mnnu.edu.cn (Z. Cai).

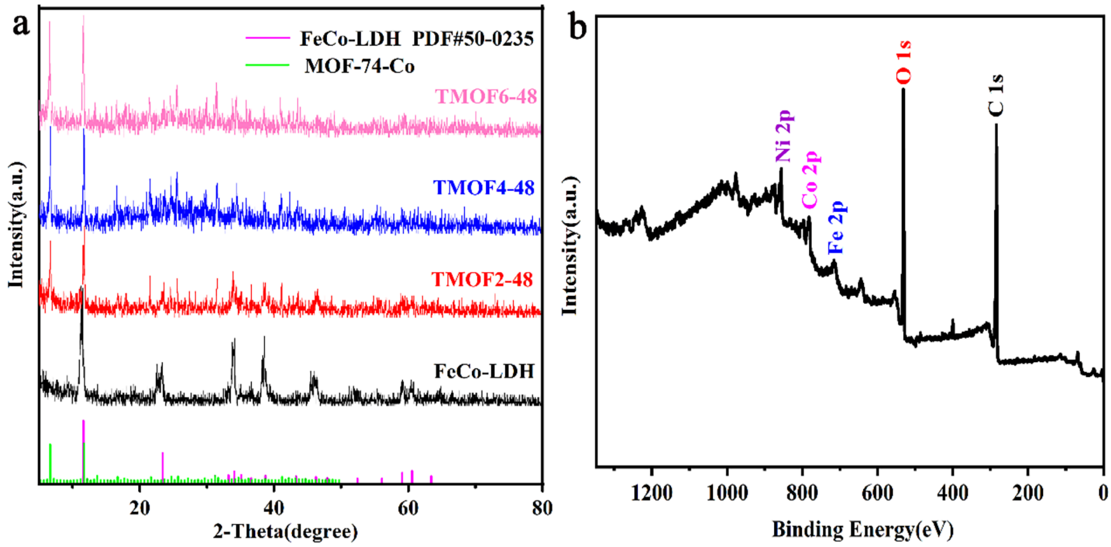

Fig. S2 (a) XRD patterns of FeCo-LDH, TMOF2-48, TMOF4-48 and TMOF6-48. (b) XPS survey spectra of the obtained TMOF4-48.

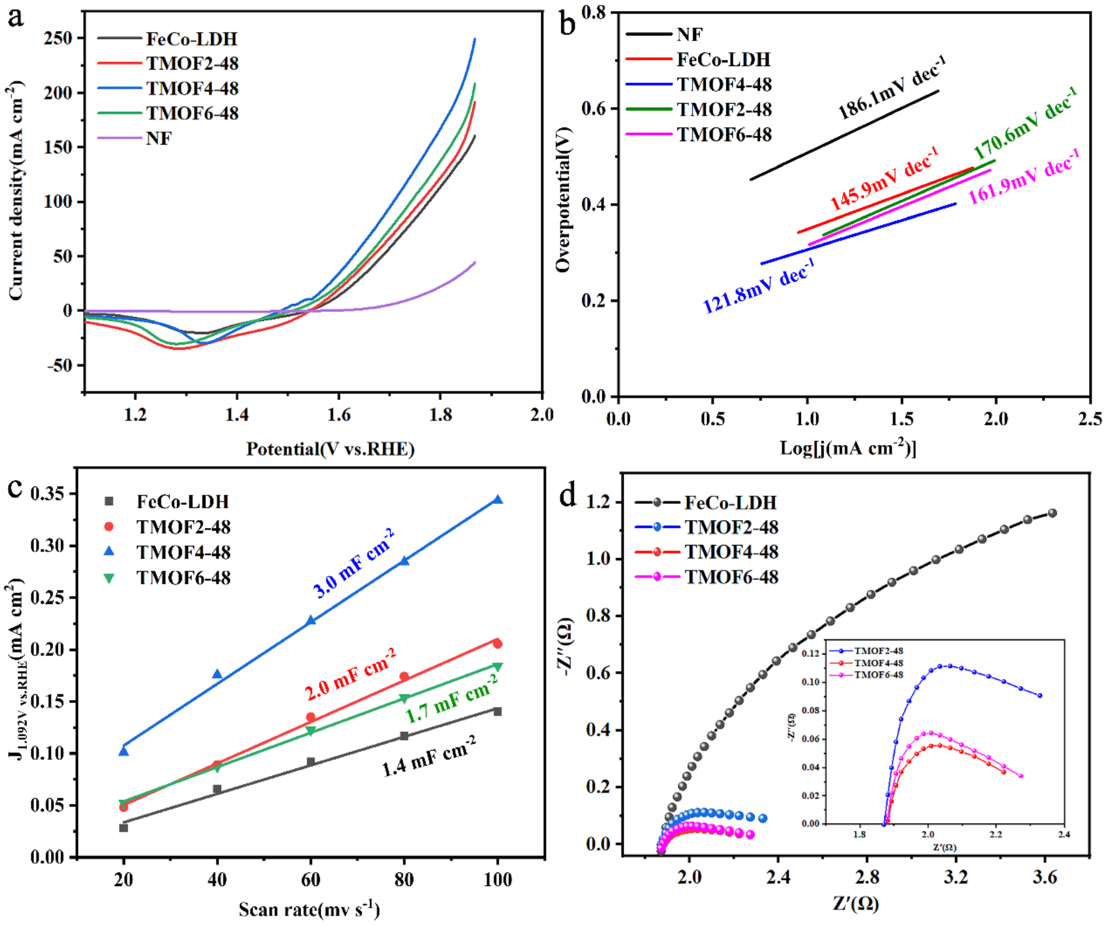

Fig. S3 (a) Polarization curves and (b) The corresponding Tafel plots of OER on the FeCo-LDH, TMOF2-48, TMOF4-48, TMOF6-48 and NF. (c) C_{dl} values of FeCo-LDH, TMOF2-48, TMOF4-48 and TMOF6-48. (d) EIS spectra of prepared catalysts in 1.0 M KOH.

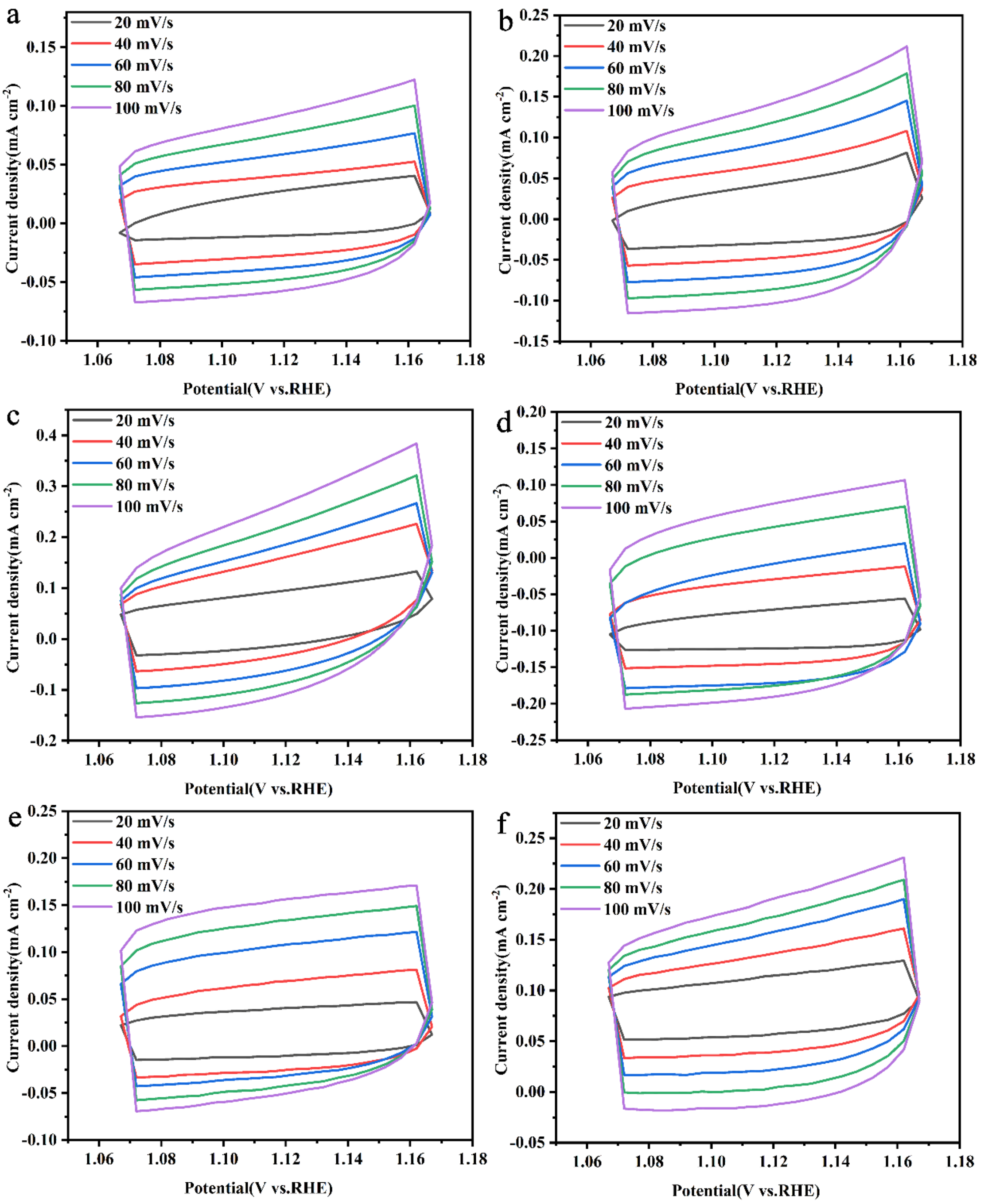

Fig. S4 Cyclic voltammograms of (a) FeCo-LDH, (b) TMOF4-24, (c) TMOF4-48, (d) TMOF4-72, (e) TMOF2-48 and (f) TMOF6-48 in 1.0 M KOH solutions at various scan rates within a potential range of 1.07-1.17 V.
